# Improving the quality of medical records

**DOI:** 10.1093/eurpub/ckac131.372

**Published:** 2022-10-25

**Authors:** G Guarducci, I Sanguineti, C Cuccaro, R Randisi, G Messina, N Nante

**Affiliations:** 1 Post Graduate School of Public Health, University of Siena, Siena, Italy; 2 San Michele Private Hospital, Albenga, Italy; 3 Department of Molecular and Developmental Medicine, University of Siena, Siena, Italy; 4 Legal Affair Unit, Regional Technical-Administrative Support Agency - ESTAR, Florence, Italy

## Abstract

**Background:**

Medical record is an essential tool both in patients’ diagnostic and therapeutic pathways and communication between different care providers. It also has an economic-administrative, medical-legal and epidemiological function. From an economic-administrative point of view, a medical record allows an evaluation and review of services to better manage the corporate health budget. In addition, it allows traceability and complete transparency of the health activities carried out. The study evaluates the formal quality of medical records compiled in an Italian private clinic before and after a training intervention.

**Methods:**

In June 2019, a retrospective study was carried out to assess a private clinic’s quality of medical records. One month later, healthcare providers were trained on the appropriate compilation of medical records, whose pre-printed format was structurally improved. In March 2020, we verified the quality of medical records produced after that training intervention. Statistical analysis (Wilcoxon test) was carried out through Stata.

**Results:**

A total of 149 medical records were reviewed. Statistically significant improvements (p < 0,05), between before and after training intervention, were for overall readability (33.3% vs 74.7%), completeness of admission and discharge dates (33.3% vs 74.40%), for completeness of anamnesis (13.6% vs 63.9%), for completeness of therapeutic card (53% vs 85.5%), in the reduction of non-compliance corrections (22.7% vs 4.8%), signature presence of physical examination (34.9% vs 71.1%) and for signature presence in the hospital discharge card (86.4% vs 96.4%).

**Conclusions:**

The results show that simple measures, such as staff training and restructuring of the format, are effective in improving the quality of medical records.

**Key messages:**

• Healthcare providers should perceive the proper completion of medical records as a common goal.

• Well-completed medical records contribute to better health care.

